# Developing of Granulomatosis with Polyangiitis during Etanercept Therapy

**DOI:** 10.1155/2014/210108

**Published:** 2014-03-06

**Authors:** María Clara Ortiz-Sierra, Andrés Felipe Echeverri, Gabriel J. Tobón, Carlos Alberto Cañas

**Affiliations:** ^1^Department of Internal Medicine, Fundación Valle del Lili, CES University Medicine School, Medellín, Colombia; ^2^Unit of Rheumatology, Fundación Valle del Lili, ICESI University Medicine School, Carrera 98 18-49, Cali, Colombia

## Abstract

We describe a 67-year-old woman who developed c-ANCA positive vasculitis with involvement in eyes, skin, kidney, peripheral nerves, and upper and lower airway during treatment with etanercept therapy for rheumatoid arthritis. A diagnosis of Granulomatosis with Polyangiitis was done. Thus, anti-TNF therapy may be associated with the development of ANCA positive vasculitis.

## 1. Introduction

The tumor necrosis factor alpha (TNF-*α*) is a proinflammatory cytokine involved in the pathogenesis of many inflammatory and autoimmune conditions as rheumatoid arthritis (RA) and inflammatory bowel disease among others. Several anti-TNF-*α* biological agents have been approved for the management of RA [1]. The most frequent adverse events related to anti-TNF-*α* therapies are infectious diseases, malignancies, demyelinating diseases, and drug-induced lupus [2, 3]. In addition, treatment with etanercept has been associated with the development of diverse granulomatous diseases such as sarcoidosis [4–6], granulomatous hepatitis [7], granulomatous thyroiditis [8], and Wegener's granulomatosis (now denoted as Granulomatosis with Polyangiitis-GPA-) [9]. Here, we report a new case of GPA developing in a patient with RA treated with etanercept.

## 2. Case Report

**Figure 1:**
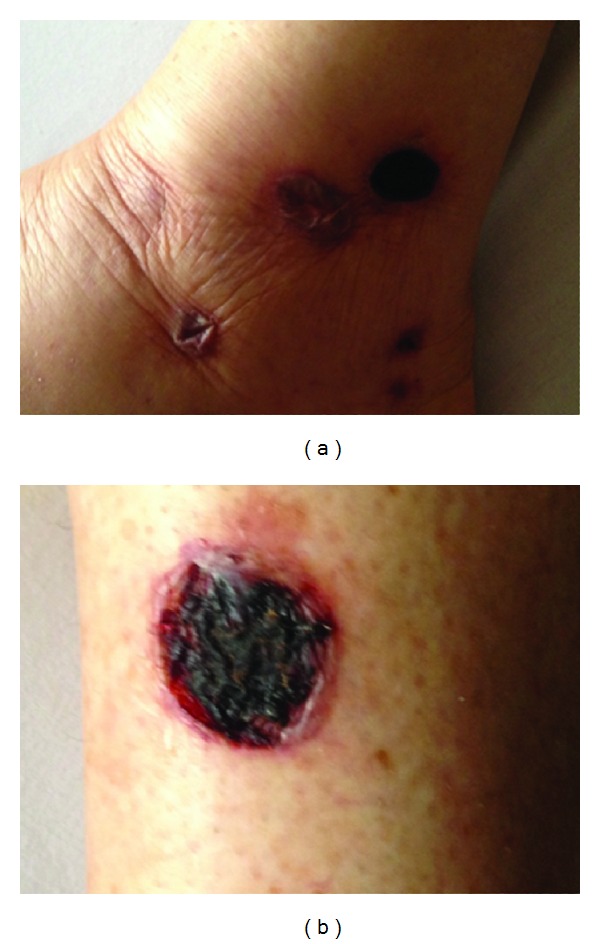
Vasculitic lesions with necrotic center in lower limbs in a patient with granulomatosis with polyangiitis.

**Figure 2:**
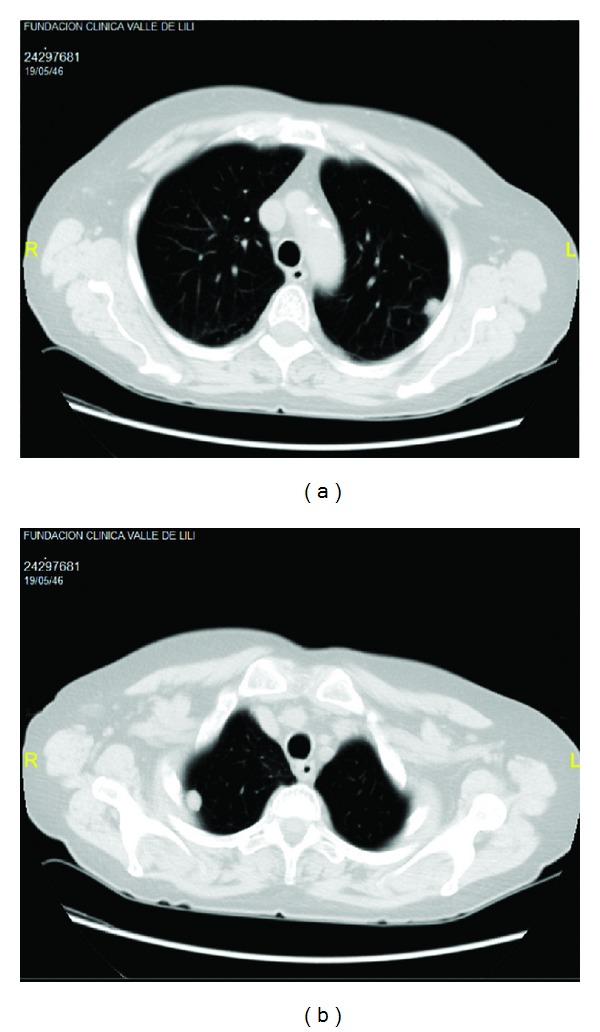
Pulmonary nodules seen on CT imaging of a patient with granulomatosis with polyangiitis.

**Figure 3:**
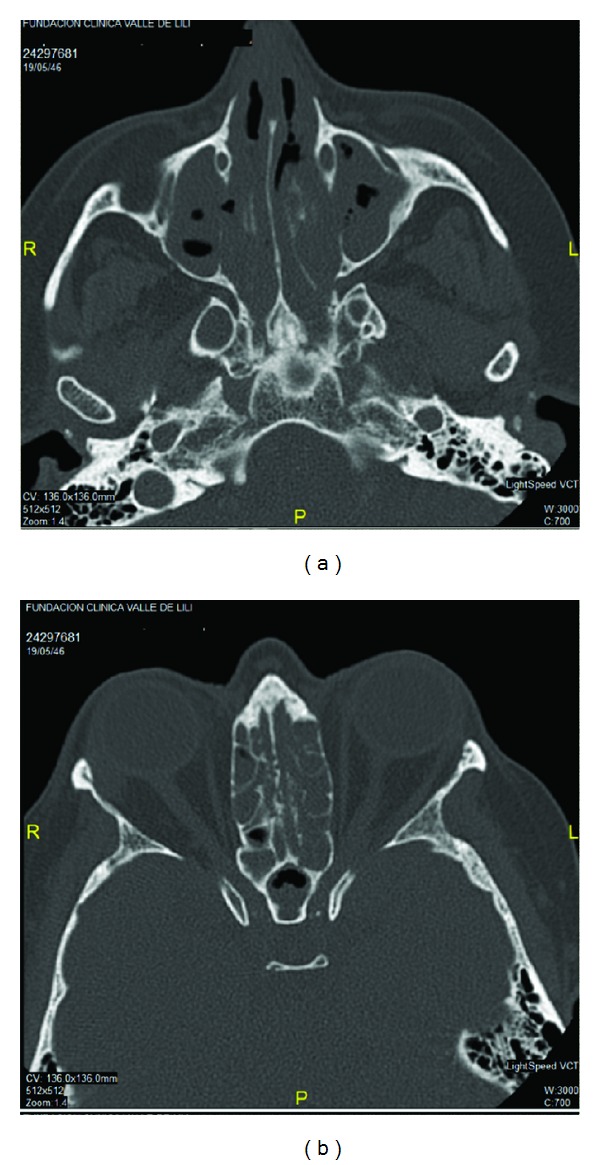
CT showing a diffuse sinus disease without erosions in a patient with granulomatosis with polyangiitis.

A 67-year-old woman was admitted in our service by a clinical picture of two weeks of new onset-very painful and erythematous nodules in lower limbs that were followed by ulceration. She additionally manifested constitutional symptoms, conjunctive injection, nasal congestion, paresthesia, and paresia in the lower limbs associated with peripheral neuropathy and polyarthritis. She had a 2-year history of RA refractory to treatment with prednisolone (10 mgr/day) and methotrexate (15 mgr/weekly). After 8 months of this treatment and because of high activity disease score, a treatment with subcutaneous etanercept (50 mg weekly) was initiated three months before admission. The physical examination at admission revealed bilateral conjunctive injection, and the ophthalmologic exam showed bilateral scleritis. Deformity in hands with synovitis and nodules in the right elbow extensor aspect was also observed. Ulcerated lesions with necrotic center in lower limbs were evident (Figure 1). Laboratory test showed normal hemoglobin (13 g/dL), high WBC, and platelets count (18,000/mL and 542,000/mL, resp.). No lymphopenia was found. Acute phase reactants were elevated, with an erythrosedimentation rate of 111 mm/hour and C-reactive protein of 15.3 mg/dL (normal <0.5 mg/dL). Renal and liver functions were normal. The autoantibodies profile showed positivity for Rheumatoid Factor (45 IU/mL, cut-off 14 IU/mL), antinuclear antibodies (1 : 320 homogeneous), and c-ANCA (1/320). The urinalysis showed hematuria without other relevant pathological findings. Multiple pulmonary parenchymal subpleural nodules were observed in chest radiography and CT (Figure 2). Additionally paranasal sinus CT was done showing chronic sinusopathy without bone erosions or cartilage destruction (Figure 3). A skin biopsy was performed showing inflammatory lesions with necrotic center consistent with leukocytoclastic vasculitis. A clinical GPA diagnosis was done. Treatment with methylprednisolone pulses and cyclophosphamide 750 mg monthly was started, with subsequent steroid tapering regimen. At 3-month follow-up the patient presented a good clinical response with complete improvement of necrotic lesions, decrease in pulmonary nodules, and symptoms of upper airway.

## 3. Discussion

GPA (formerly named as Wegener's granulomatosis) is a systemic granulomatous disease associated with small and medium vessel vasculitis of unknown origin that primarily affects the upper respiratory tract, lungs, glomeruli, skin, kidneys, and peripheral nerves. The diagnosis is established based on clinical, radiological findings, presence of c-ANCA (by immunofluoresce), and anti-Proteinase 3 (PR3) antibodies by ELISA, supported by histological evidence of granulomas and vasculitis [10]. There is evidence that granuloma and vasculitis formation occurs in a genetically susceptible host as a result of autoimmune phenomena. Some experimental studies show that the c-ANCA (anti-PR3) antibodies are important to granuloma formation and chronic/refractory vascular involvement. Although the etiology of this disease is unknown, the most implicated environmental target is the bacterial infections, implicating* S. aureus* superantigens or Gram-negative bacteria [11]. In addition, some pharmacological molecules, including antithyroid drugs, may induce ANCA positivity. Also TNF-*α* is implicated in the GPA development. This cytokine plays a critical role in the immune system development and response regulation. Indeed, TNF-*α* stimulation primed the PR3 expression in mononuclear cells. This activation is needed to start the physiopathological axis in vasculitis.

Although different to the expected action of TNF-*α* in vasculitis, with the extended use of anti-TNF-*α* agents in diverse clinical scenarios, rare adverse events, including granulomatous diseases such as sarcoidosis and GPA, have been described. Thus, granulomatous diseases may be triggered or worsened by anti-TNF therapies [8, 9]. Anti-TNF-*α* may additionally trigger systemic autoimmune diseases as systemic lupus erythematosus [3].

Differences in the mode of neutralizing TNF-*α* action between these TNF-*α* antagonist may be implicated in the genesis of granulomatous reaction. Etanercept, a biologic agent composed of two recombinant forms of the human TNF receptor P75 fused to an Fc portion of human immunoglobulin G1, blocks primordially soluble TNF-*α* and has poor effect on tissue TNF-*α*. The effect of Etanercept over dilution of granulomas seems to be less important that those induce by antibodies against TNF-*α* such as infliximab that block this cytokine both tissue and soluble forms [12].

Although less expected as an adverse event, GPA development has been described with anti-TNF-*α* therapies such as etanercept [9] and golimumab [13]. However, the underlying mechanism of granulomatous conditions after anti-TNF-*α* exposure is not fully explained. Cytokine misbalance after TNF-*α* inhibition may induce granuloma formation in a compensatory way [4, 14].

In conclusion, we reported a new case of GPA developing during etanercept therapy. This clinical observation must be considered in all patients treated with anti-TNF.
